# Impact of Single-Melamine Tautomerization on the Excitation of Molecular Vibrations in Inelastic Electron Tunneling Spectroscopy

**DOI:** 10.1021/acs.nanolett.4c00904

**Published:** 2024-05-15

**Authors:** Manex Alkorta, Rebecca Cizek, Nicolas Néel, Thomas Frederiksen, Jörg Kröger

**Affiliations:** † Donostia International Physics Center (DIPC), E-20018 Donostia-San Sebastián, Spain; ‡ Centro de Física de Materiales (CFM) CSIC-UPV/EHU, E-20018 Donostia-San Sebastián, Spain; ¶ Institut für Physik, Technische Universität Ilmenau, D-98693 Ilmenau, Germany; § IKERBASQUE, Basque Foundation for Science, E-48011 Bilbao, Spain

**Keywords:** Molecular vibrations, single molecule, inelastic electron tunneling spectroscopy, scanning tunneling microscopy, density functional theory, nonequilibrium transport calculations

## Abstract

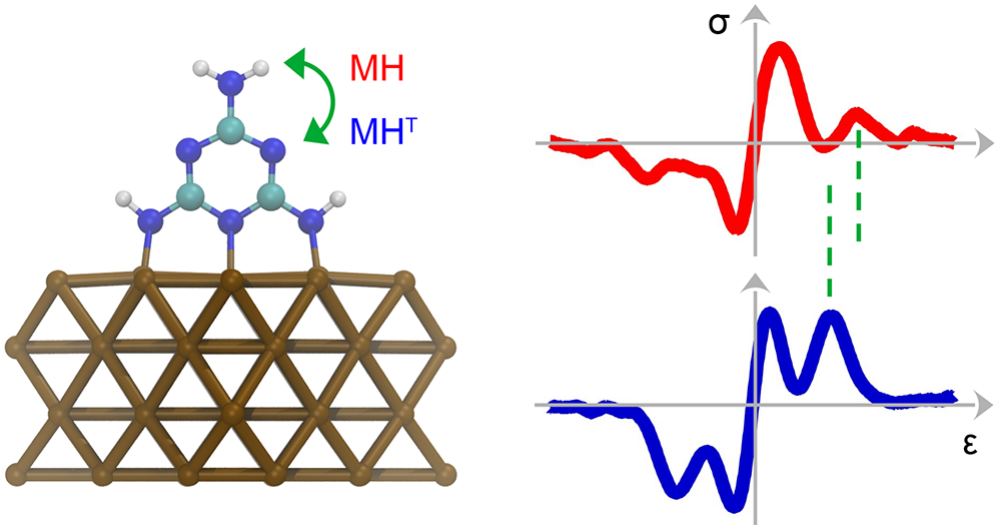

Vibrational quanta of melamine and its tautomer are analyzed at the single-molecule level on Cu(100) with inelastic electron tunneling spectroscopy. The on-surface tautomerization gives rise to markedly different low-energy vibrational spectra of the isomers, as evidenced by a shift in mode energies and a variation in inelastic cross sections. Spatially resolved spectroscopy reveals the maximum signal strength on an orbital nodal plane, excluding resonant inelastic tunneling as the mechanism underlying the quantum excitations. Decreasing the probe–molecule separation down to the formation of a chemical bond between the melamine amino group and the Cu apex atom of the tip leads to a quenched vibrational spectrum with different excitation energies. Density functional and electron transport calculations reproduce the experimental findings and show that the shift in the quantum energies applies to internal molecular bending modes. The simulations moreover suggest that the bond formation represents an efficient manner of tautomerizing the molecule.

Inelastic electron tunneling spectroscopy (IETS) was pioneered in early experiments using planar tunneling junctions.^1−4^ The suitability of a scanning tunneling microscope (STM) for inelastic excitations was suggested^5^ and evidenced by graphite phonon spectroscopy.^6^ In a seminal work, IETS was then carried out at the single-molecule level with an STM complementing its atomic-scale characterization with chemical identification.^7^ A wealth of experimental reports on single-atom and single-molecule vibrational spectroscopy with an STM followed, which has been summarized by a few review articles.^8−13^

The applicability of selection rules in IETS with an STM is both appealing and challenging. Propensity rules take the symmetry of vibrational modes and orbitals of the adsorbed atom or molecule close to the Fermi energy (*E*_F_) into account for describing magnitude and sign of the conductance changes induced by the quantum excitation.^14−18^ These propensity rules successfully explained the occurrence of only the C–H stretch mode out of the entire vibration spectrum of C_2_H_4_ on Cu(100) in IETS.^7,14,15^ In these approaches, the tip electronic structure is considered as an *s*-wave function within the Tersoff–Hamann approximation.^19,20^ A more general theoretical picture of IETS considers the symmetry of both tip and sample orbitals as well as the symmetry of vibrational modes to understand conductance changes due to quantum excitation in experimental IETS.^21^ Indeed, tip effects were previously shown to be important for the interpretation of IETS data.^21−26^ For instance, in vibronic spectroscopy of pentacene on ultrathin insulating films, it was shown that the signal strength in vibrational progression, i.e., in orbital replica due to on-resonance vibration excitation within the Franck–Condon mechanism, depends on the local symmetry of molecular and tip orbitals.^25,27^ However, experimental reports on the applicability of the underlying theoretical picture,^21^ particularly in off-resonance spectroscopy, are very scarce.^28^ Therefore, benchmarking state-of-the-art simulations and the hitherto theoretical validity of selection rules against experimental spectroscopy of quantum vibrations is one of the main motivations of the presented work. Moreover, on-surface reactions at the single-molecule level including the microscopic and spectroscopic characterization of the educt and product are relevant to nanotechnological engineering and functionalization of surfaces.

The findings reported here unveil the intimate relationship between active molecular vibrations in off-resonance STM-IETS and the symmetry of the most transmitting electron transport channels of the entire junction. To achieve these results, single melamine (1,3,5-triazine-2,4,6-triamine, C_3_H_6_N_6_, MH) molecules were adsorbed on Cu(100) and studied in IETS experiments. The tautomers MH^T^ exhibit shifted mode energies and different inelastic signal strengths compared with MH in the spectra. Accompanying transport calculations rationalize these observations in terms of the local symmetry character of both the most transmitting eigenchannels of the junction and the deformation potential associated with the excited bending vibrational modes of melamine. Importantly, these conclusions are distinctly different from a previous vibronic spectroscopy experiment,^25^ where the vibration-assisted overlap of tip and molecular orbitals determined the excitation strength in resonant tunneling. Indeed, experimental and calculated spatially resolved IETS data for MH clearly demonstrate the off-resonance character of the vibrational excitations. Collapsing the tunneling barrier by forming a chemical bond between the foremost Cu atom of the tip and the top amino group of MH quenches the inelastic signal and changes the vibrational energies, which hints at the concomitant tautomerization, as supported by the calculations.

Figure 1 summarizes the topographic (Figure 1a,b) and spectroscopic (Figure 1c) data of MH and MH^T^ adsorbed on Cu(100). In agreement with previous observations,^29,30^ the STM data of MH exhibit *C*_2*v*_ symmetry with a linear depression separating two oval protrusions. The latter are due to molecular frontier π-orbitals, whose nodal planes coincide with the molecular backbone. The relaxed adsorption geometries are depicted for MH in Figure 1d and for MH^T^ in Figure 1e. Upon adsorption, two amino groups of the molecule lose a H atom each, thereby enabling a chemical bond of three N atoms with the Cu(100) surface. The resulting adsorption geometry is an upright-standing molecule.^29^ A flat adsorption geometry, i.e., the adsorption of the triangular plane of the molecule parallel to the surface, was previously observed only for low-temperature (6 K) deposition.^29^

**Figure 1:**
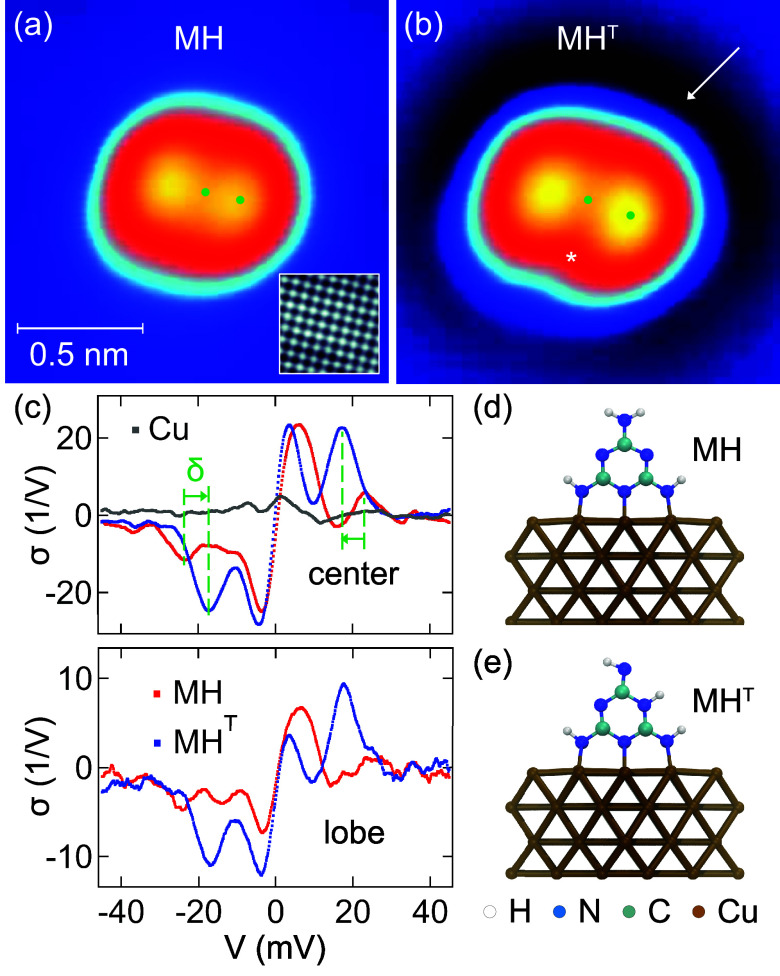
Topographic and spectroscopic data of melamine and its tautomer on Cu(100). (a) STM image of MH (sample voltage: 100 mV, tunneling current: 50 pA, size: 1.5 nm × 1.5 nm). Dots indicate spectroscopy positions. Inset: constant-height map (10 mV, 2.1 nm × 2.1 nm) of the tunneling current obtained with a CO-terminated tip, showing the atomically resolved Cu(100) lattice (the feedback loop had been disabled at 100 mV and 50 pA prior to ramping the tip by 300 pm toward the surface). (b) As (a), for MH^T^. The asterisk marks the tautomerization site, and the arrow marks a dark rim encircling the molecule. (c) IETS data acquired atop the center (top) and a lobe (bottom) of MH and MH^T^, as indicated by the dots in (a) and (b). The shift of a spectroscopic signature upon tautomerization is indicated by δ. A representative vibrational spectrum of clean Cu(100) is depicted as gray dots. The feedback loop was disabled at 75 mV and 80 pA for all spectra. (d, e) Relaxed adsorption geometries of MH and MH^T^.

Injecting tunneling electrons at an elevated sample voltage exceeding 2.4 V with the tip positioned above the molecular plane induces a tautomerization, where one H atom of the top amino group is transferred to a neighboring N atom, in accordance with a previous report^29^ and theoretical analysis.^31^ The STM image of the product molecule MH^T^ exhibits a reduced symmetry that consists of only a single mirror plane coinciding with the molecular backbone (Figure 1b). The orbital nodal plane now appears as a nonuniform depression with the site of missing H (asterisk in Figure 1b) being darker than the site with remaining H. Another remarkable feature in the STM images of the tautomers is the dark rim surrounding MH^T^ (arrow in Figure 1b), which is absent for MH. Its dependence on the bias voltage is presented in the Supporting Information (Figure S1). This observation is reminiscent of similar results reported for tetracyanoethylene on Ag(100),^32^ which was traced to charge transfer from the surface to the molecule and the concomitant depletion of the electron local density of states (LDOS) around the molecule. The tautomerization process is reversible, i.e., applying a voltage pulse above 2.4 V restores the educt molecule and removes the dark rim. The Supporting Information (Figure S2) summarizes similar findings for deuterated melamine (1,3,5-triazine-2,4,6-triamine-d_6_, C_3_D_6_N_6_, MD) and its tautomer MD^T^ on Cu(100).

The molecules and their tautomerized partners exhibit strong signals in IETS at low bias voltage (Figure 1c), while spectra for |*V*| > 50 mV (not shown) are essentially featureless. The spectrum of clean Cu(100) (gray dots in Figure 1c) does not reveal excitations in the relevant voltage range. For facilitated comparison with calculations, the IETS signal is defined as σ ≡ (d^2^*I*/d*V*^2^)/(d*I*/d*V*) (*I*: tunneling current, *V*: sample voltage). The σ-spectra of MH exhibit pronounced dip–peak pairs. At low bias voltage, MH exhibits a dip in σ at (−6 ± 1) mV and a peak at (4 ± 1) mV. At higher voltage, another dip–peak pair appears at (±23 ± 1) mV. Owing to the symmetric position of dips and peaks with respect to zero bias voltage, the dip–peak signatures are assigned to molecular vibrational modes with energies *ℏω*_*i*_ = |*eV*_*i*_| (*ℏ* = *h*/2π: reduced Planck constant; *e*: elementary charge; *i* = 1, 2). The simulations discussed below characterize these modes in detail. The tautomerization leaves the dip–peak pair at low voltage essentially invariant, while the pair at ±23 mV is converted into a pair at (±17 ± 1) mV for MH^T^, giving rise to a redshift of δ ≈ 6 mV. This dip–peak pair exhibits an increased signal strength compared to the pair at ±23 mV. The deuterated molecules MD and MD^T^ behave similarly (Figure S2).

Importantly, the inelastic signal strength is significantly reduced in σ-spectra acquired atop the π-orbital lobes of MH and MH^T^ (bottom spectral data in Figure 1c). Spatially resolved spectra with an increased number of sampling points along symmetry directions are presented in the Supporting Information (Figure S3). These results demonstrate that for the inelastic excitation of the low-energy vibrational quanta, resonant tunneling through molecular orbitals^33−42^ does not apply. In particular, an increased residence time of the resonant tunneling electron at the molecule and its thereby enhanced coupling to the molecular vibrational degrees of freedom are not required for the efficient excitation of vibrational quanta. The IETS data likewise reveal that at the tautomerization site (asterisk in Figure 1b), the overall signal strength for the inelastic excitations is reduced.

It is noteworthy that for MH and MD, similar σ-spectra were observed; that is, an isotope shift is absent. Therefore, it is likely and will be clarified by the simulations that the vibrational excitations underlying the inelastic signals do not involve vibrational stretch modes, which would be subject to an isotope shift due to the exchange of H by D. A recent tip-enhanced Raman spectroscopy experiment unveiled changes in the N–H stretch mode for MH and MH^T^ at elevated energies exceeding 400 meV.^30^

In order to understand the experimentally observed IETS data, transport simulations were performed with a model tip above MH and MH^T^ on Cu(100), using TranSiesta(43,44) for the electronic structure together with Inelastica(57−60) for vibrational modes and IETS (see the Theoretical Method section for details). Figure 2a,b shows constant-height electron transmission probability maps at *E*_F_ of the two isomers in the tunneling range. Consistent with the experiments and previous transport calculations,^29^ the images reveal characteristic features of the π-orbitals with maximum transmission above the lobes on either side of the molecular backbone. The simulation for MH^T^ also reveals a slight asymmetry with reduced transmission over at the tautomerized side. Figure 2c,d presents the calculated IETS data obtained with the tip positioned above the center and the lobe of the respective isomer. For MH, three groups of active vibrational modes can be distinguished, namely the frustrated translation (FT) with energy *ℏω*_FT_ = 4 meV, the frustrated rotation (FR) with *ℏω*_FR_ = 17 meV, and a set of transverse internal bending (IB) modes with *ℏω*_IB_ ≥ 23 meV (Figure S5). The calculated signals of the FR-mode and IB-mode contribute to a resulting central peak at ∼20 meV (arrows in Figure 2c). The MH^T^ isomer exhibits the FT-mode at nearly the same energy as that observed for MH, while the FR-mode appears at ℏω_FR_^T^=12 meV and IB vibrations occur with energies ℏω_IB_^T^ ≥ 17 meV. The IETS peak observed at the lowest bias voltage (|*V*_1_| = *ℏω*_1_/*e*) in the experimental spectra of both MH and MH^T^ is therefore assigned to the excitation of the FT-mode. The second mode (|*V*_2_| = *ℏω*_2_/*e*) in the experiments can be identified with an IB-mode that is redshifted in the experiments as well as in the simulations by 6 meV. Because of the finite energy resolution in the experiments, the contribution of the FR-mode cannot be excluded. Besides the reproduced redshift δ in the calculations, the simulations likewise show the reduction of the IETS signal strength at the molecular lobes (Figure 2d) compared to the molecular plane (Figure 2c), albeit to a stronger extent than in the experiments (Figure 1c).

**Figure 2:**
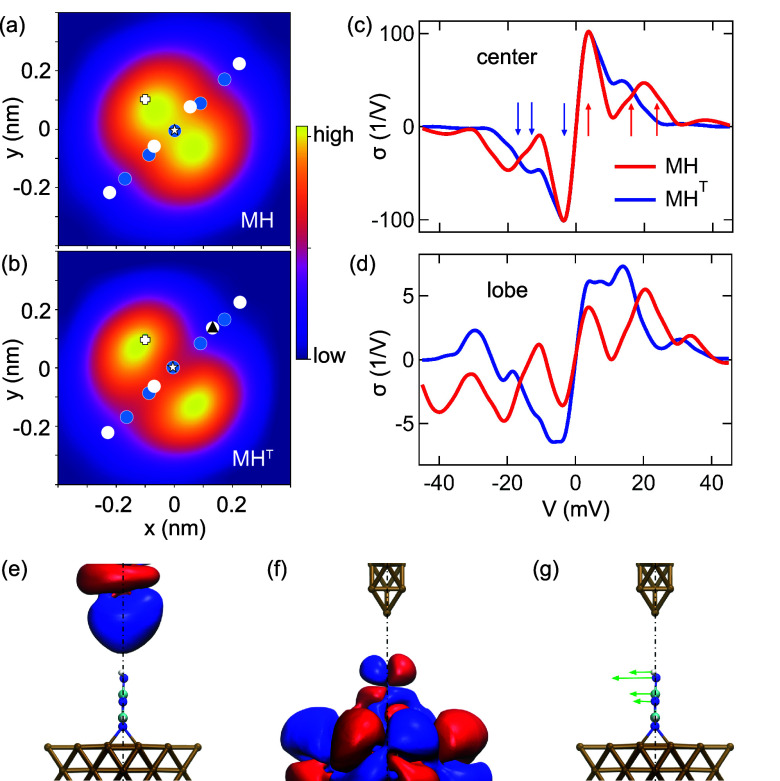
Calculated electron transport properties and IETS propensity rules for melamine and its tautomer on Cu(100) at tunneling range. (a, b) Constant-height electron transmission probability at *E*_F_ across (a) MH and (b) MH^T^ (tip height above surface of 1.1 nm corresponds to tip excursion of Δ*z* = −30 pm; linear color scale covering transmissions from 0 to (a) 1.90 × 10^–6^, (b) 0.98 × 10^–7^). The tautomerized H atom is marked with a triangle. (c) Calculated σ-spectra for the tip positioned above the center of MH and MH^T^ (star in (a) and (b)). Arrows (red for MH, blue for MH^T^) mark mode energies referred to in the text. (d) As (c), for the tip positioned above the lobes (cross in (a) and (b)). (e) Isosurface of tip *s*-wave eigenchannel scattering state near *E*_F_. (f) As (e), for molecule *p*-wave eigenchannel scattering state, reminiscent of the LUMO π-orbital resonance (Figure S4). (g) Side view of the atomic displacement pattern of the molecular FT-mode, enabling scattering of electrons between the states in (e) and (f) owing to the appropriate symmetry character with respect to the transport axis (dash-dotted line).

The analysis of eigenchannel scattering states of the junction^45^ explains why these modes are active in IETS. The two most important scattering states for electrons close to *E*_F_ are depicted in Figure 2e,f. At this tip height, which belongs to the far tunneling range, the interaction between molecule and tip apex is sufficiently weak for inhibiting the molecule from tilting, resulting in well-defined symmetries with respect to the transport axis (dash-dotted line). Electrons incoming from the tip with *s*-wave character decay slowly toward the molecule in the vacuum barrier, while electrons from the molecule predominantly exhibit *p*-wave character (Figure S4), mostly reflecting the lowest unoccupied molecular orbital (LUMO) resonance. Despite the symmetry mismatch between tip and molecule orbitals, electron transport can occur in the presence of inelastic processes.^18^ To this end, molecular vibrational quanta with the appropriate symmetry (odd with respect to inversion through the transport axis) must be excited by the tunneling electrons. The atomic displacement patterns of the FT-mode (Figure 2g), FR-mode, and IB-mode (Figure S5) are suitable in this sense. When the tip is positioned above one of the orbital lobes, no strict symmetries are present, and the propensity rules are relaxed. As the electron scattering states in this situation are locally rotationally symmetric around an axis connecting the tip apex and molecular lobe, the overall IETS signal of the transverse modes is substantially reduced, reflecting the deviation from the ideal case of scattering between *s*-wave and *p*-wave states.

In additional experiments, changes in IETS were probed as a function of the vertical tip–molecule distance across the vacuum barrier (Figure 3). The junction conductance *G* = *I*/*V* was gradually increased from tunneling to contact and beyond by displacing the tip toward the molecule. The conductance variation *G*(Δ*z*) (Δ*z*: tip displacement) exhibits the expected uniform exponential increase for 0 ≤ Δ*z* ≤ Δz*. At Δz* ≈ 232 pm, *G* changes from 3 m*G*_0_ to 8 m*G*_0_ (*G*_0_ = 2*e*^2^/*h*: quantum of conductance) in an abrupt manner on the time scale of data acquisition. This clear deviation from an exponential variation of *G* signals the formation of a chemical bond between the tip and the molecule.^46,47^ For Δ*z* > Δz*, *G* remains nearly constant. The formation of the contact junction is reversible and not detrimental to either the tip or the MH structural integrity, as verified by comparison of STM images before and after the contact. At the tip displacements Δ*z* indicated in Figure 3b, IETS was performed. Two effects can be inferred from the spectra. First, the overall IETS signal strength is reduced when the tip approaches the tunneling-to-contact transition (Δ*z* ≥ 200 pm). Second, the energy *ℏω*_2_ decreases from 23 to 17 meV for these tip excursions, while a possible change in *ℏω*_1_ falls below the detection limit. The decrease in *ℏω*_2_ to 17 meV matches the shift of this mode upon tautomerization. Therefore, it is likely that contact formation entails tautomerization. The simulations discussed below indeed reveal that the total energy of the tip–MH^T^ complex dives below the total energy of the tip–MH complex at these tip excursions. Upon retraction of the tip, the MH is restored, as observed from STM images and IETS data. Contact experiments for MH^T^ were impeded due to junction instabilities. Indeed, the chemical bond formation between the Cu-terminated tip and the two tautomers differs, which was explored in atomic force microscopy experiments and will be the focus of a forthcoming publication.

**Figure 3:**
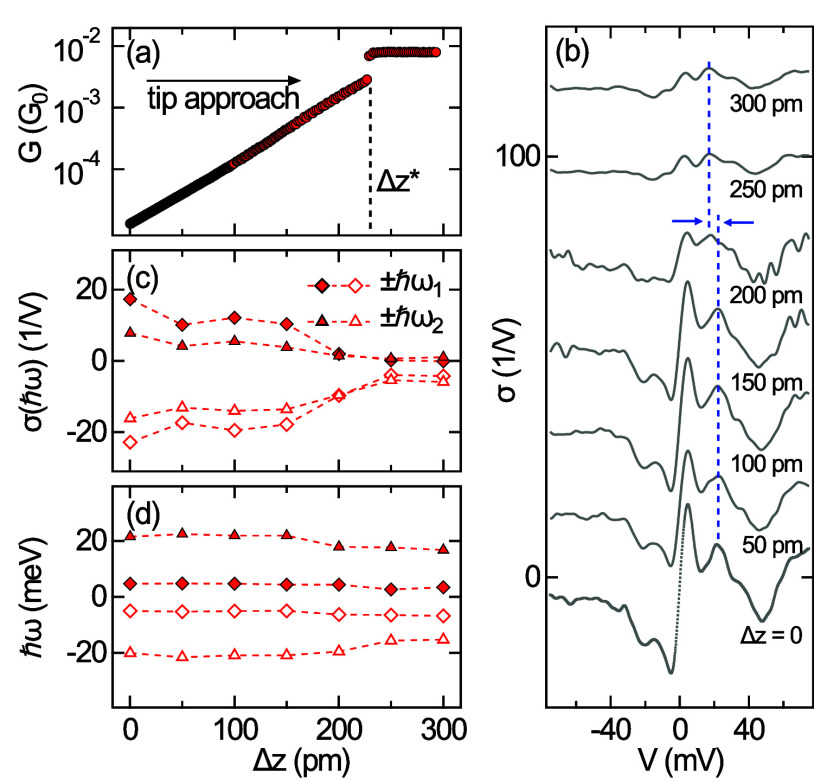
Transition from tunneling to contact for MH. (a) Conductance (*G*) versus tip displacement (Δ*z*) with Δ*z* = 0 defined by feedback loop parameters 75 mV and 75 pA and Δz* ≈ 232 pm marking the abrupt transition from tunneling (Δ*z* < Δz*) to contact (Δ*z* > Δz*). (b) Collection of σ-spectra acquired at the indicated tip displacements with marked shift of a vibrational signature (dashed lines). For all spectra, the feedback loop was disabled at 75 mV, and the tip was displaced by Δ*z*. (c) Variation of σ(*ℏω*_*i*_) (*i* = 1, 2) as a function of Δ*z*. (d) As (c), for *ℏω*_*i*_. The legend to (c) applies to (d) as well. The dashed lines in (c) and (d) are guides for the eye.

The observed experimental trends of a reduction of the IETS signal and a redshift of specific vibrational energies with increasing junction conductance are reproduced by the calculated σ-spectra of MH (Figure 4a) and MH^T^ (Figure 4b). In the far tunneling range (Δ*z* ≤ 0), the overall IETS signal of MH is reduced with increasing tip excursion due to a weak tilting of the molecule and the concomitant mismatch between the transport axis and the molecular orbital nodal plane (Figure S6). For MH^T^, in contrast, the attractive interaction with the tip retains the orientation of the molecular plane down to contact distances. Therefore, the MH^T^ junction exhibits a stronger overall IETS signal than MH.

**Figure 4:**
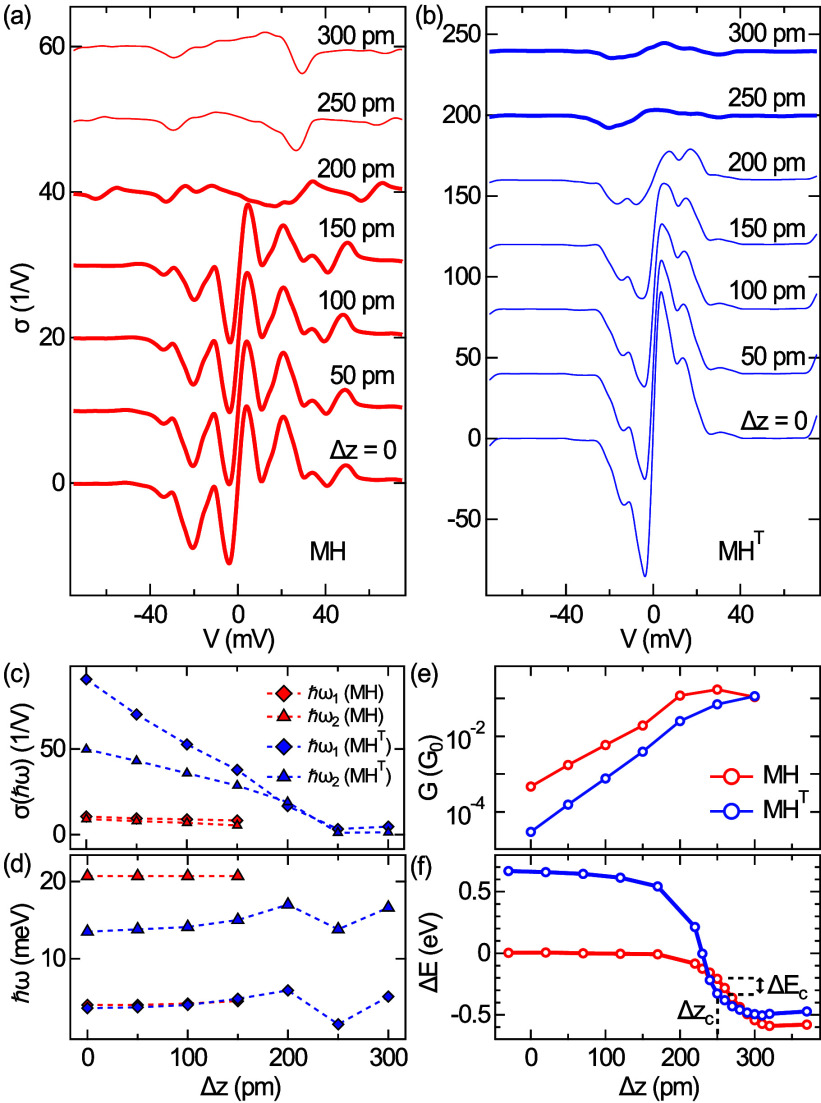
Simulated IETS data depending on the tip–molecule distance. (a) Spectra of σ for MH depending on the indicated tip excursion Δ*z*. (b) As (a), for MH^T^. Thick (thin) lines mark the data for the energetically preferred isomer (MH for 0 ≤ Δ*z* < 200 pm, MH^T^ for Δ*z* > 200 pm). (c) Variation of the IETS signal strength of excited vibrational modes as a function of Δ*z*. (d) As (c), for the excitation energies *ℏω*. (e) Evolution of the junction conductance for MH and MH^T^ with Δ*z*. (f) Interaction energy of tip–isomer hybrid relative to the tip–MH energy as a function Δ*z*. The maximum energy difference is |Δ*E*_c_| = 0.12 eV at Δ*z*_c_ = 250 pm, which belongs to the contact range (see (e)).

In addition to this qualitative difference between the two tautomers, a quenching of the signal strength with tip approach was found. To see this quenching and the variation in vibrational energies more clearly, the peak heights of the quantum excitations (Figure 4c) and their energies (Figure 4d) are displayed separately. Figure 4e depicts the evolution of junction conductance *G* with the tip excursion for MH and MH^T^. As in the experiments, the onset of contact, i.e., the collapse of the tunneling barrier, is indicated by the deviation of *G* from an exponential variation. The overall lower conductance of the MH^T^ junction follows from a reduction of electronic states at *E*_F_ that belong to the LUMO, which compared to MH is shifted to higher energies *E* > *E*_F_ (Figure S7). At contact (Δ*z* > 200 pm), the calculated junction conductance is ∼0.1*G*_0_, which is larger than the experimental observations. Based on these results, the additional gradual reduction of the IETS signal with decreasing junction width can be explained as follows.^18,33^ Approaching the tip toward the molecule, the LUMO energy is reduced with respect to *E*_F_ (Figures S8–S10), which increases the elastic electron transport at the expense of the IETS signal strength. Concomitantly with decreasing tip–molecule separation, the coupling of the molecule to the tip becomes similar to the molecule–surface coupling, which likewise tends to weaken the IETS cross section (Figure S11).

The simulations likewise offer a rationale for the apparent redshift of the IB-mode upon bond formation with the tip. Figure 4f compares the interaction energies of the tip and molecule for MH and MH^T^. Intriguingly, in the range of chemical bond distances (Δ*z* ≈ Δ*z*_c_) between the tip apex Cu atom and the top amino group of the respective isomer, tautomer MH^T^ is preferred. Therefore, based on these simulations, the following scenario can be proposed. The tautomerization reaction can be induced due to the proximity of the tip. At contact, the molecular junction comprises MH^T^ with a redshifted IB vibrational mode. Upon retraction of the tip, MH becomes the preferred isomer again (Figure 4f), and the tautomerization is reversed.

In conclusion, the reversible tautomerization of a single melamine molecule on Cu(100) induced by local electron injection imposes changes in the molecular low-energy vibrational spectrum. The combination of experimental spectroscopic data and nonequilibrium transport calculations unveils that the frustrated translation is unaffected by the reaction, while a melamine internal bending mode is subject to an ample redshift. Collapsing the tunneling barrier between the tip and melamine weakens the inelastic scattering cross section owing to an enhanced elastic electron transport channel, which is caused by the LUMO shifting slightly toward the Fermi level and the additional hybridization with the tip. The concomitant redshift of the internal bending mode acts as a litmus test for the tautomerization of melamine owing to the bond formation between the tip and the molecule. The presented studies therefore reveal the appealing control of on-surface single-molecule reactions and the theory-supported spectroscopic characterization of the reactants. The findings are relevant to fields as diverse as nanotechnology, molecular electronics, and functionalized architectures at surfaces.

## Experimental Method

The experiments were performed with an STM operated in an ultrahigh vacuum (10^–9^ Pa) and at low temperature (5 K). Surfaces of Cu(100) were cleaned and prepared by Ar^+^ ion bombardment and annealing. The clean surface was exposed at room temperature to MH sublimated from a powder (purity: 98%) in a heated (320 K) Ta crucible. A chemically etched W wire (purity: 99.95%, diameter: 125 μm) served as the tip material. Field emission on and repeated indentations into the Cu surface presumably led to the coating of the tip apex with the substrate material. Single-atom transfer from the tip to the sample gave rise to particularly sharp and stable probes.^46−51^ The CO molecules for tip decoration were adsorbed at low temperature (6 K) from the gas phase (purity: 99.97%) with a partial pressure of 10^–7^ Pa. The transfer of a single CO molecule from the surface to the tip followed a standard routine.^52^ Topographic data were acquired in the constant-current mode with the bias voltage applied to the sample and were further processed with WSXM.^53^ Spectroscopy of d*I*/d*V* and d^2^*I*/d*V*^2^ proceeded via the sinusoidal modulation (5 mV_rms_, 350 Hz) of the dc sample voltage and measuring the first and second harmonics, respectively, of the ac current response of the tunneling junction with a lock-in amplifier.

## Theoretical Method

The density functional calculations for MH and MH^T^ are based on a 3 × 3 Cu(100) surface cell in TranSiesta,^43,44^ the transport module of Siesta(54) within the Generalized Gradient Approximation.^55^ A double-zeta plus polarization basis was used for the molecular species (C, H, and N) as well as the Cu apex atom of the tip, and a single-zeta plus polarization atomic basis was used for the remaining Cu atoms. Pseudopotentials and corresponding basis radii were taken from the SIMUNE Atomistics data set.^56^ The real-space grid was defined by a 400 Ry energy cutoff. In the transport setup, periodicity along the transport direction is replaced by self-energies, while the transverse (surface) directions are sampled on a 3 × 3 *k*-grid for the 2D Brillouin zone. Elastic transmissions were computed with Tbtrans on a 10 × 10 *k*-mesh, while eigenchannels, vibrational modes, electron–phonon couplings, and IETS spectra were obtained with Inelastica(57−60) at the Γ point, employing a finite-difference scheme with a 2 pm displacement amplitude for the molecule and tip apex atoms only, a voltage fraction factor of 0.25 over the substrate–molecule interface, and experimental broadening corresponding to a sample voltage modulation of 5 mV_rms_ and a temperature of 4.2 K.

## Supporting Information Available

Voltage-dependent topographies of melamine, microscopies and spectroscopies of deuterated melamine, spatially resolved spectroscopy of melamine, transmission eigenchannels, active vibrational modes in inelastic electron tunneling, magnitude of inelastic conductance change from DFT, transmission function and density of states, Fermi level alignment with molecular resonances, and two-level model for sign and magnitude of inelastic conductance change (PDF)
