# Supplementary material for: Impact of Single-Melamine Tautomerization on the Excitation of Molecular Vibrations in Inelastic Electron Tunneling Spectroscopy
Source: Nano Lett. 2024 May 15;24(24):7195–201. doi: 10.1021/acs.nanolett.4c00904 (PMC11194823; doi:10.1021/acs.nanolett.4c00904)
Supplement: Supplementary file 1 — nl4c00904_si_001.pdf [file nl4c00904_si_001.pdf]

Supporting Information

Impact of single-melamine tautomerization on the excitation of molecular vibrations in inelastic electron tunneling spectroscopy

Manex Alkorta,[†,‡,‖] Rebecca Cizek,[¶,‖] Nicolas Néel,[¶] Thomas Frederiksen,[†,§] and Jörg Kröger[*,¶]

†*Donostia International Physics Center (DIPC), E-20018 Donostia-San Sebastián, Spain*

‡*Centro de Física de Materiales (CFM) CSIC-UPV/EHU, E-20018, Donostia-San Sebastián, Spain*

¶*Institut für Physik, Technische Universität Ilmenau, D-98693 Ilmenau, Germany*

§*IKERBASQUE, Basque Foundation for Science, E-48011 Bilbao, Spain*

‖*These authors contributed equally.*

E-mail: joerg.kroeger@tu-ilmenau.de

Voltage-dependent topographies of melamine

The dark rim encircling MH^T in STM images is further analyzed here in topographic data acquired at different bias voltages (Figures S1a–d). Cross-sectional profiles along the direction indicated in Figure S1a by the dashed arrow show that the apparent depth of the rim increases with increasing bias voltage (Figure S1e). At the same time the apparent height and width of the molecule decrease.

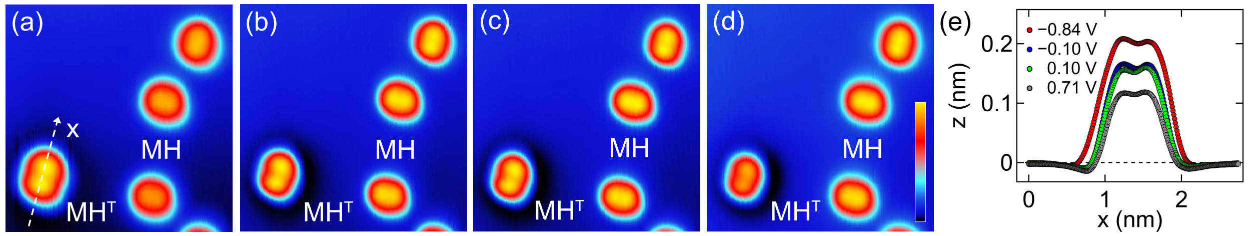

Figure S1: Topographic STM data of MH and MH^T at different bias voltages. The images were acquired at (a) -0.84 V, (b) -0.10 V, (c) 0.1 V, (d) 0.71 V and 110 pA (4.1 nm × 4.1 nm). (e) Cross-sectional profiles of MH^T recorded along the path x indicated in a.

Deuterated melamine

Figure S2 shows that deuterated melamine (1,3,5-triazine-2,4,6-trianine-d_6, $C_3D_6N_6$, MD) and its tautomer MD^T exhibit similar properties as observed for MH and MH^T. The intact MD molecule appears with C_{2v} symmetry in STM images (Figure S2a), where a linear depression signals an orbital nodal plane that coincides with the upright standing molecular backbone and that separates two oval protrusions related to frontier π-orbitals. Tautomerization of MD is achieved by electron injection through the center of the nodal line at elevated sample voltage (≥ 2.4 V). The resulting tautomer exhibits mirror symmetry with respect to the molecular plane (Figure S2b). Similar to observations from MH^T, the tautomerization site of MD^T (asterisk in Figure S2b) appears with a reduced apparent height. Spectra of $\sigma = (\mathrm{d}^2I/\mathrm{d}V^2)/(\mathrm{d}I/\mathrm{d}V)$ acquired atop the center of the linear depression are depicted in Figure S2c. For MD, vibrational excitations show their signatures at $(\pm 4 \pm 1)$ mV and

($\pm20 \pm 1$) mV. Tautomerization leaves the dip-peak pair at ($\pm4 \pm 1$) mV nearly invariant, while the higher-voltage signature shifts to ($\pm16 \pm 1$) mV for MD^T.

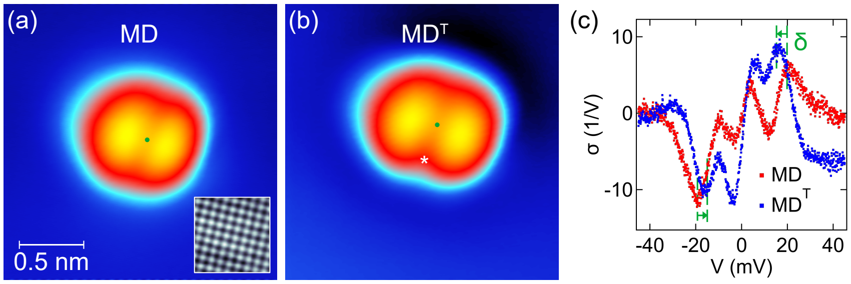

Figure S2: Topographic and spectroscopic data of deuterated melamine (MD) and its tautomer (MD^T) on Cu(100). (a) STM image of MH (50 mV, 50 pA, 2.1 nm × 2.1 nm). Inset: Constant-height map (10 mV, 2.1 nm × 2.1 nm) of the tunneling current obtained with a CO-terminated tip (the feedback loop had been disabled at 100 mV and 50 pA prior to ramping the tip by 300 pm toward the surface) showing the atomically resolved Cu(100) lattice. (b) As a, for MD^T. (c) IETS data acquired atop the center of MD and MD^T, as indicated by the dots in a and b. The shift of a spectroscopic signature upon tautomerization is indicated by δ. The feedback loop was disabled at 50 mV and 65 pA.

Spatially resolved spectroscopy of melamine

Spatially resolved IETS reveals that the inelastic signal strength is largest along the orbital nodal line (arrow denoted $\parallel$ in the inset to Figure S3d), as shown for MH in Figure S3a. In contrast, the IETS signal is quickly quenched upon reaching the protruding π-orbitals along a direction perpendicular to the nodal line and crossing the molecular center (arrow denoted $\perp$ in the inset to Figure S3d), as can be inferred from the collection of spectra in Figure S3b. To see these changes more clearly, the signal strength $\sigma(\hbar\omega_i)$ ($i = 1, 2$) is plotted for the MH vibrational modes with energies $\hbar\omega_1$ and $\hbar\omega_2$ as a function of $\ell_\parallel$ (Figure S3c) and $\ell_\perp$ (Figure S3d), which denote the distances covered by the paths parallel and perpendicular to the linear depression, respectively.

While $\sigma(\hbar\omega_i)$ shows clear variations along the $\parallel$ and $\perp$ paths across the molecule, the energies $\hbar\omega_i$ stay virtually constant (Figure S3e,f). A similar situation is encountered for MH^T (Figure S3g–l) with a notable exception. The variation of σ with $\ell_\parallel$ (Figure S3i)

is clearly asymmetric with σ increasing slowly toward the MH^T center ($\ell_{\parallel} < 0.6$ nm) and decreasing fast for $\ell_{\parallel} > 0.6$ nm.

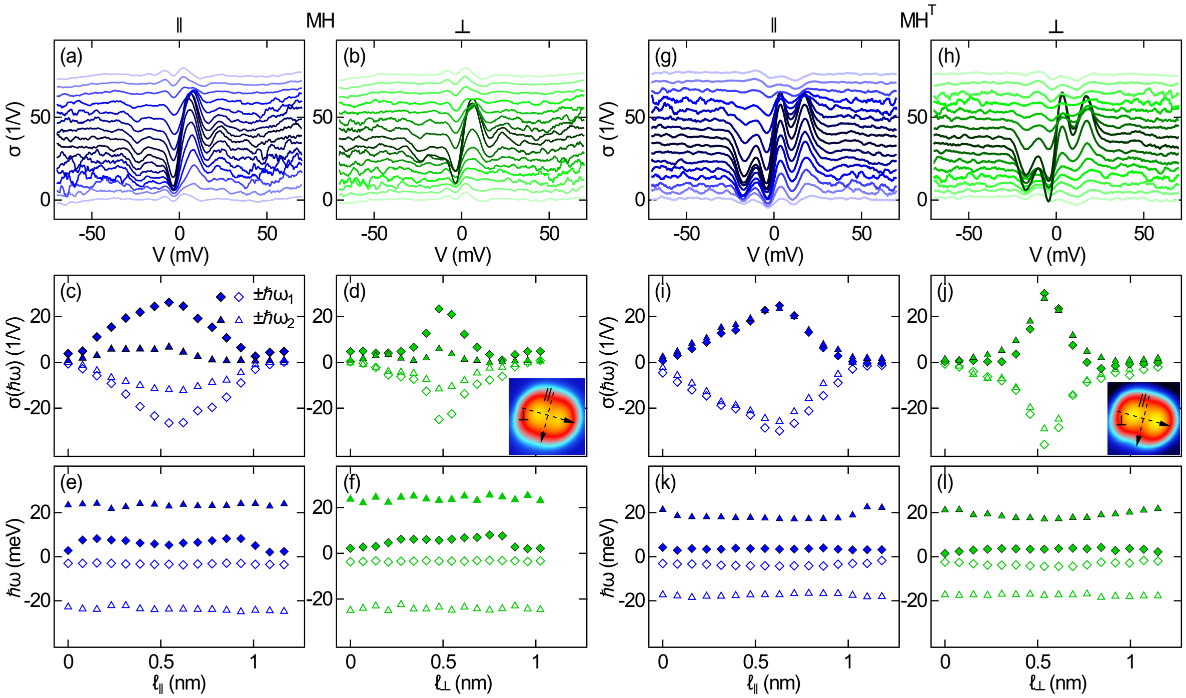

Figure S3: Spatially resolved IETS of MH and MH^T. (a) Collection of spectra parallel ($\parallel$) to the orbital nodal line of MH ($\ell_{\parallel} = 0$ coincides with the upper end of the arrow ($\parallel$) in panel d). (b) As a, for a direction perpendicular ($\perp$) to the orbital line across the MH center ($\ell_{\perp} = 0$ coincides with the left end of the arrow ($\perp$) in panel d). (c) Variation of σ as a function of $\ell_{\parallel}$ for vibration excitations with energies $\hbar\omega_1$ and $\hbar\omega_2$. (d) As c, for $\ell_{\perp}$. (e) Variation of $\hbar\omega_1$ and $\hbar\omega_2$ with $\ell_{\parallel}$. (f) As e, for $\ell_{\perp}$. (g)–(l) As a–f, for MH^T. Inset to panel d,j: STM image of (d) MH and (j) MH^T (100 mV, 50 pA, 1 nm × 1 nm) with indicated directions $\parallel$ and $\perp$. The legend in c applies to d–f and i–l. Feedback loop parameters for all spectra: 75 mV, 500 pA.

Transmission eigenchannels

Figure S4 depicts the main transmission eigenchannels for the MH junction in the tunneling range ($\Delta z = -30$ pm ensuring the upright orientation of the MH plane) at different energies: close to the highest occupied molecular orbital (HOMO) resonance (Figures S4a,b), at the Fermi level (Figures S4c,d), and at the lowest unoccupied molecular orbital (LUMO) resonance (Figures S4e,f).

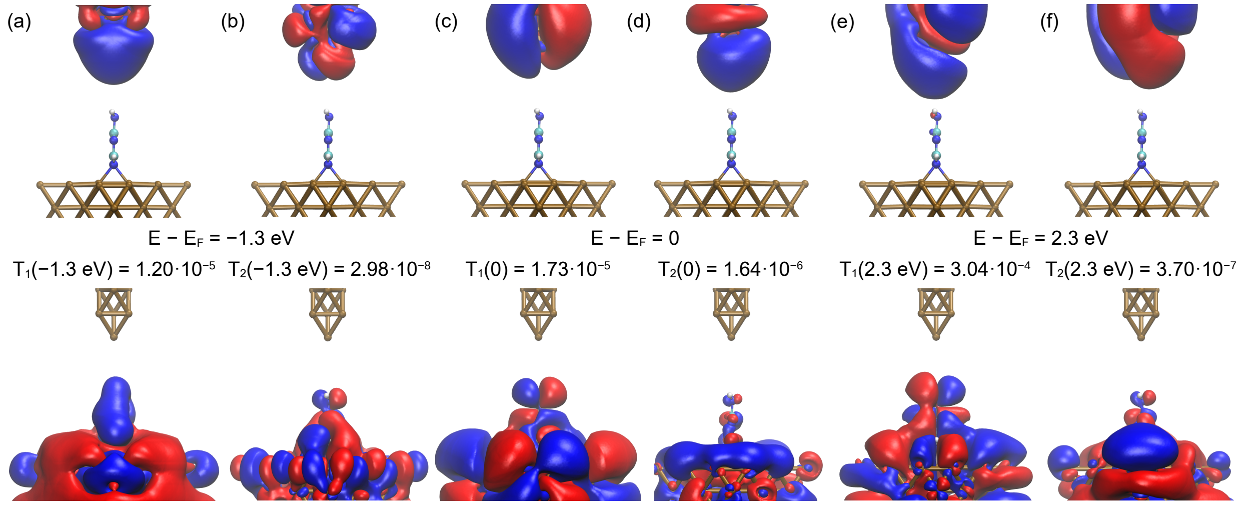

Figure S4: Isosurfaces of the scattering states for the main transmission eigenchannels of MH at characteristic energies in the tunneling range ($\Delta z = -30\,\text{pm}$). (a),(b) Near the HOMO resonance at $E - E_\text{F} = -1.3\,\text{eV}$. (c),(d) At the Fermi energy E_F. (e),(f) Near the LUMO resonance at $E - E_\text{F} = 2.3\,\text{eV}$. The top (bottom) row shows scattering states incoming from the tip (substrate). Energies $E - E_\text{F}$ and transmission probabilities T_1, T_2 of the eigenchannels are indicated. Panel c, substrate, and panel d, tip, are presented in Figure 2 of the article.

Two eigenchannels are dominant for transport in the vicinity of E_F (Figures S4c,d). Electrons coming from the substrate predominantly reach the top of the molecule through a p-wave LUMO-type scattering state, while electrons coming from the tip predominantly reach the molecule with s-wave character. Therefore, the inelastic contribution to the total current is dominated by scattering between tip s-wave and surface p-wave scattering states (and vice versa) while exciting a vibration quantum in the molecular junction. This mismatch in orbital symmetry explains why transverse vibrational modes with an odd symmetry with respect to inversion through the transport axis are active in IETS. The isosurface plots of the eigenchannels at the energies of the frontier molecular orbitals (Figures S4a,b,e,f) further suggest that the p-wave character observed in topographic and vibrational spectra of MH reflects the tail of the LUMO resonance at E_F.

Active vibrational modes in inelastic electron tunneling

Only a few vibrational modes of MH and MH^T are active in IETS and contribute a signal to the σ-spectra (Figure S5). These modes are classified in three groups: the frustrated translation (FT) with lowest energy, the frustrated rotation (FR) with the second lowest energy, and the transverse internal bending modes (IB), which represent a set of low-energy modes obeying the required symmetries. For both isomers, the second dip–peak pair in the σ-spectra is a combination of FR-modes and IB-modes.

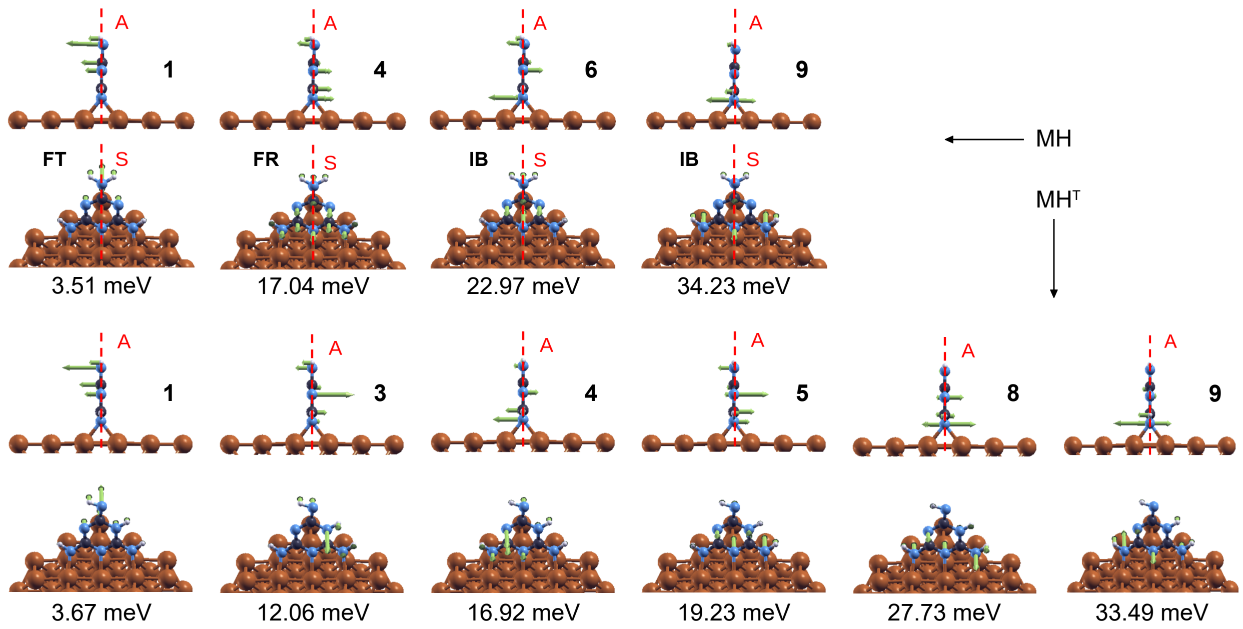

Figure S5: Low-energy vibrational modes of MH (top row) and MH^T (bottom row) that are active in IETS, calculated at a tip excursion of $\Delta z = -30\,\mathrm{pm}$. Indices 1, 4, 6, 9 (MH) and 1, 3, 4, 5, 8, 9 (MH^T) mark the energetically ordered most active vibrational modes of each isomer. The atomic displacement patterns associated with the vibrational modes can be identified as symmetric (S) and antisymmetric (A) with respect to mirror planes parallel and perpendicular to the molecular backbone. The tautomer MH^T does not exhibit a mirror plane perpendicular to its backbone.

Magnitude of inelastic conductance change from DFT

Figures S6a,b show the evolution of the mode-resolved IETS cross section Δ/T ($T = T(E)$: energy-dependent elastic transmission, $\Delta = \Delta(E)$: energy-dependent inelastic conductance correction) as a function of the tip excursion Δz for MH (Figure S6a) and MH^T (Figure S6b), which results directly from the DFT-NEGF calculations. The cross sections for MH reveal that the shoulders to the calculated IETS peak around $\pm 20\,\text{meV}$ (Figure 2c) originate from mode 4 ($\hbar\omega_4 \approx 17\,\text{meV}$) and mode 6 ($\hbar\omega_6 \approx 23\,\text{meV}$).

Figures S6c,d present the tilt angle of the two tautomers enclosed with the surface normal. For MH, at $\Delta z = -30\,\text{pm}$ the molecule is vertical and the IETS signal substantially larger than in the other tilted configurations. This enhancement of the IETS cross section at zero tilt angle can be understood by the perfect satisfaction of the symmetry selection rules.

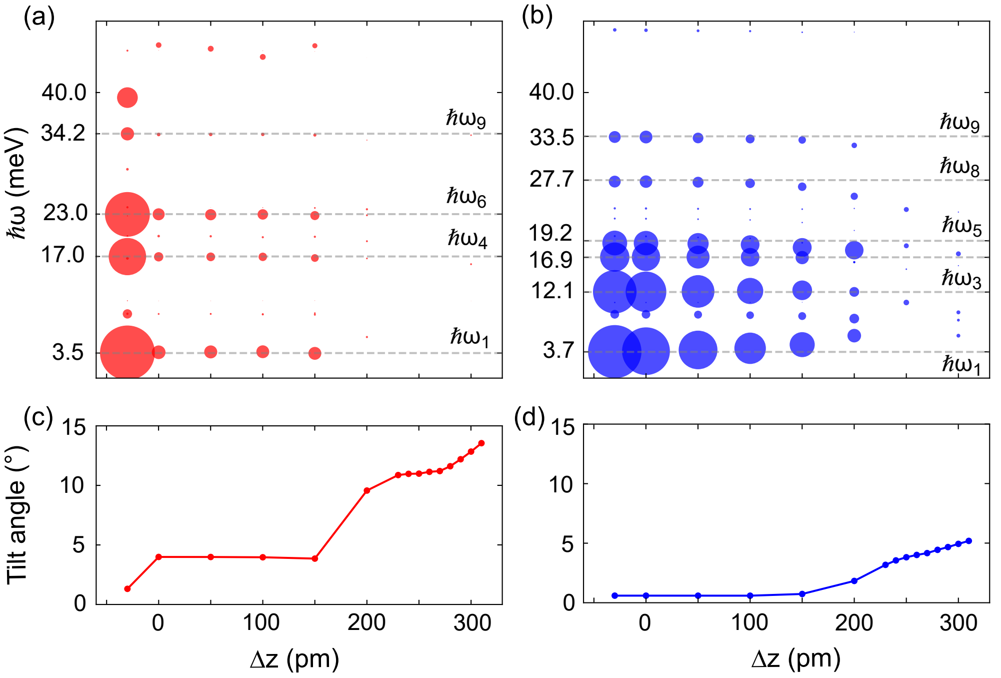

Figure S6: (a) Evolution of energies $\hbar\omega_i$ and inelastic cross sections Δ_i/T for each vibrational mode i of MH as a function of tip excursion Δz. The size of the circles is proportional to the magnitude of Δ_i/T and the dashed horizontal lines are guides to the eye for $\hbar\omega_i$ of the most IETS-active low-energy modes presented in Figure S5. (b) As panel a, for MH^T. (c) Tilt angle of MH as a function of tip excursion Δz. (d) As panel c, for MH^T.

Transmission function and density of states

Figure S7a shows the transmission functions $T(E)$ of MH and MH^T for a tip height corresponding to the tunneling range ($\Delta z = -30$ pm). For energies above -1 eV the transmission of MH clearly exceeds the transmission of MH^T, consistent with the larger conductance of the single-MH junction reported in Figure 4e of the article.

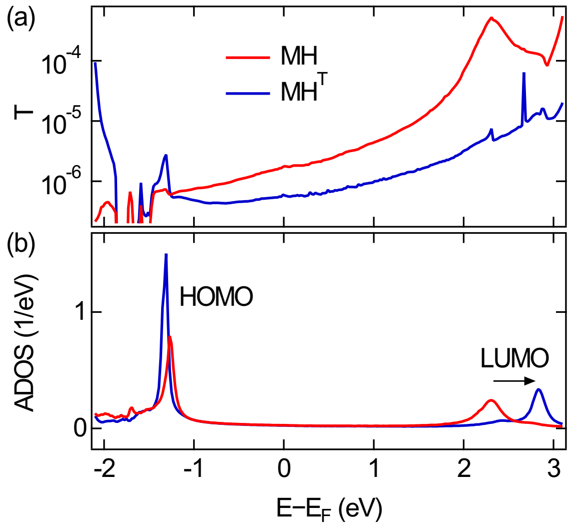

Figure S7: (a) Calculated transmission functions $T(E)$ of MH and MH^T at a tip position of $\Delta z = -30$ pm above the center of the molecule. The legend applies to panel b, too. (b) Atom-resolved density of states (ADOS) for MH and MH^T. The upshift of the LUMO resonance upon MH→MH^T tautomerization is indicated with the arrow.

The surface-electrode contribution to the atom-resolved density of states (ADOS) for the molecule (Figure S7b) relates the transmission maxima in Figure S7a to the HOMO and LUMO. The ADOS likewise offers a rationale to the larger conductance of the MH junction compared to MH^T in the simulations (Figure 4e of the article). Upon tautomerization, the LUMO of MH shifts from 2.3 eV to 2.8 eV for MH^T. This upshift of 0.5 eV significantly reduces the electron transmission of the molecular junction in a large energy window around E_F because transport is predominantly carried by the LUMO resonance. These results are consistent with previous transport simulations.[1]

Figure S8 shows the ADOS variation upon approaching the contact range. For MH no

substantial change is observed up to $\Delta z = 200\,\text{pm}$, where the LUMO resonance is shifted down from 2.3 eV to 2.1 eV. In the case of MH^T, the hybridization is much stronger due to the attraction between the top amino group and the tip. The amount of states at energies

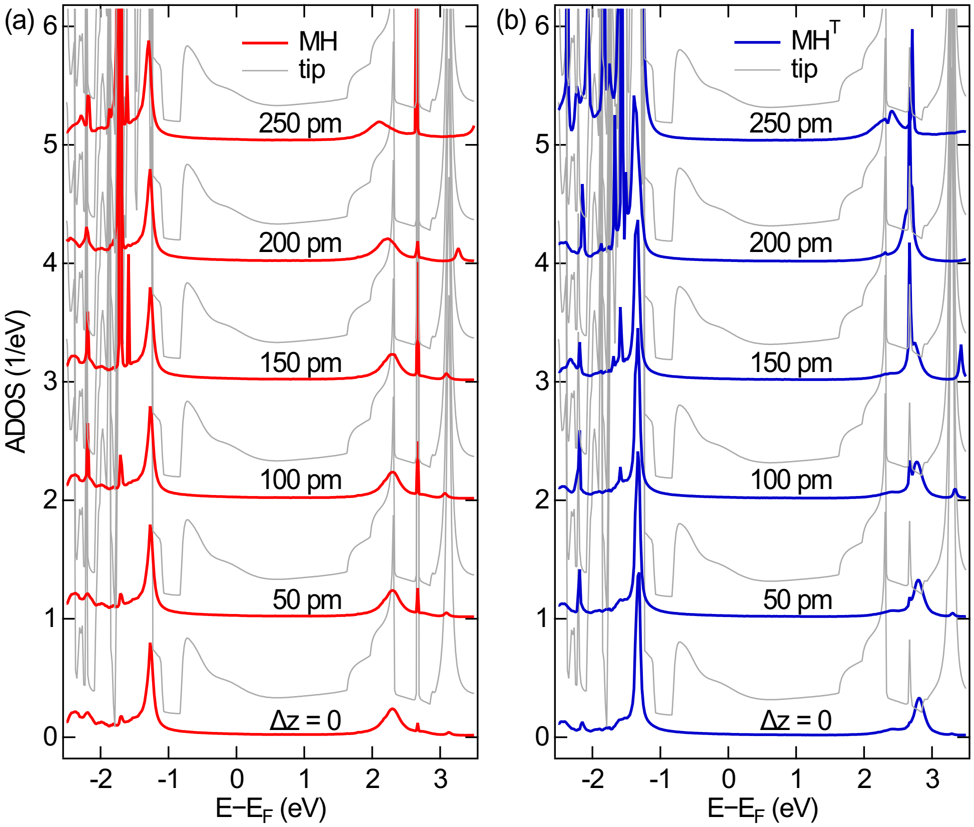

Figure S8: Substrate ADOS projected onto molecular basis orbitals from tunneling ($\Delta z = 0$) to contact ($\Delta z = 250\,\text{pm}$) separations for (a) MH in red and (b) MH^T in blue. The data are vertically offset by 1/eV. The light gray line in each panel represents the tip ADOS at $\Delta z = 0$ projected onto the 4 last apex atoms.

around 2.25 eV progressively grow from tunneling to contact ranges. When the contact is formed, the LUMO resonance of MH^T is shifted to 2.4 eV. Consequently, for MH^T the amount of reminiscent states of the LUMO resonance at E_F increases significantly (compared to MH) from tunneling to contact, thereby nearly doubling the MH^T junction transmission at E_F compared with the MH junction at contact (Figure S9).

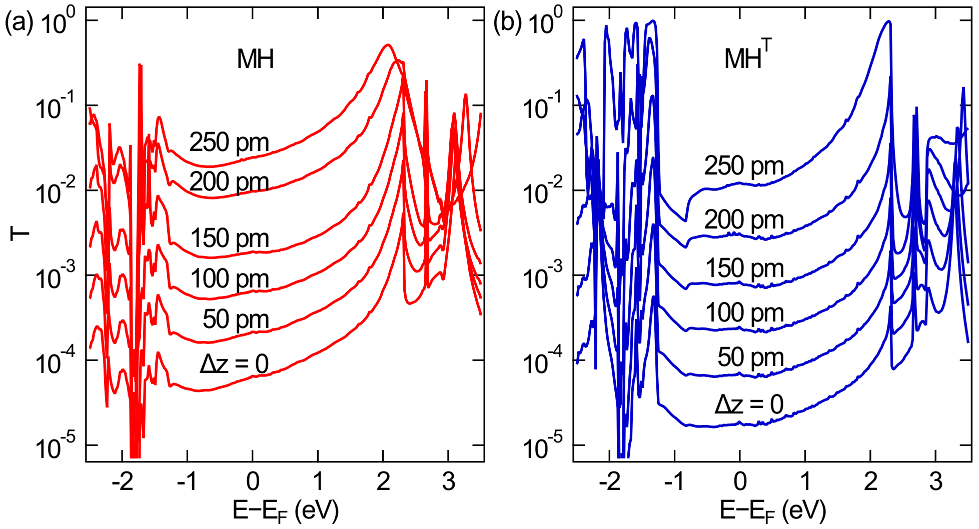

Figure S9: Transmission from tunneling ($\Delta z = 0$) to contact ($\Delta z = 250\,\mathrm{pm}$) separations for (a) MH and (b) MH^{T}.

Fermi level alignment with molecular resonances

Figure S10 demonstrates how the inelastic cross section is affected by the presence of LUMO states close to E_{F}. To this end, the Fermi level is artificially shifted relative to the molecular resonance, as can be explored in INELASTICA[2] through the optional `--Energy` parameter. The calculations presented in the article correspond to the energy reference definition $E_{\mathrm{F}} = 0$.

Two-level model for sign and magnitude of inelastic conductance change

To understand the evolution of the IETS signal from tunneling to contact ranges, it is illustrative to consider a two-site toy model defined by the following tight-binding Hamiltonian

$$H = \begin{pmatrix} \varepsilon_L & -t \\ -t & \varepsilon_R \end{pmatrix}, \tag{S1}$$

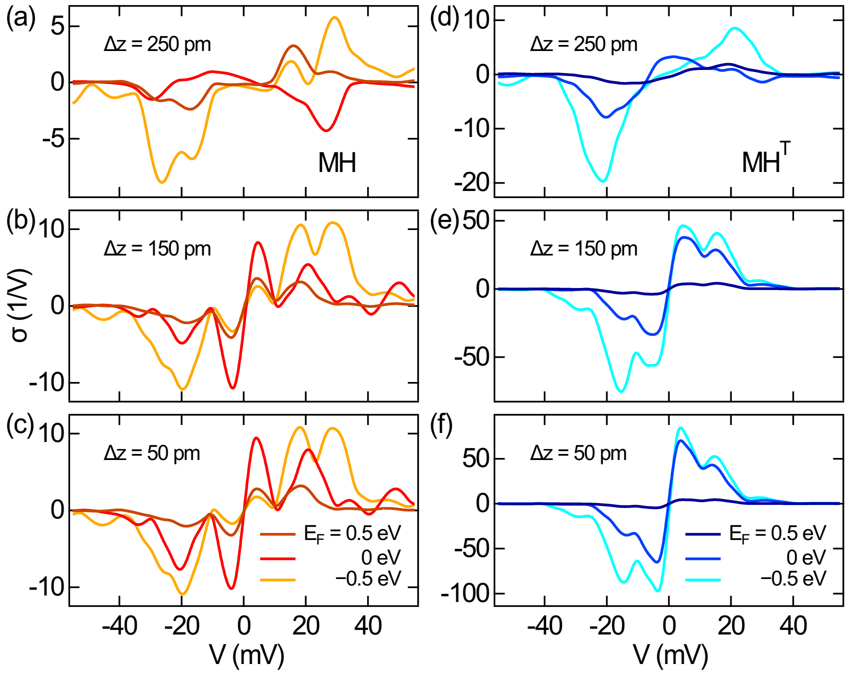

Figure S10: Calculated σ-spectra for (a)–(c) MH and (d)–(f) MH^{T} at the indicated tip excursions Δz. The Fermi level is artificially varied at each excursion with $E_{\mathrm{F}} = 0$ defined as the energy reference based on self-consistent density functional calculations. For both isomers, the signal is quenched when the Fermi level is shifted closer to the LUMO resonance.

where $\varepsilon_{L/R} = 0$ (set to zero for simplicity) are the onsite energies of left/right sites and $t > 0$ denotes the magnitude of the intersite hopping. The coupling of the molecule $\gamma_{L/R}$ to the two electrodes L/R is introduced in the wide-band limit, i. e, the tunneling rates read

$$\Gamma_L = \begin{pmatrix} \gamma_L & 0 \\ 0 & 0 \end{pmatrix} \quad \text{and} \quad \Gamma_R = \begin{pmatrix} 0 & 0 \\ 0 & \gamma_R \end{pmatrix}. \tag{S2}$$

In the energy eigenbasis, the Hamiltonian becomes

$$\tilde{H} = UHU^{\dagger} = \begin{pmatrix} \varepsilon_{\mathrm{HOMO}} & 0 \\ 0 & \varepsilon_{\mathrm{LUMO}} \end{pmatrix} \tag{S3}$$

with U the transformation U that diagonalizes H and with $\varepsilon_{\mathrm{HOMO}} = -t$ and $\varepsilon_{\mathrm{LUMO}} = +t$ the HOMO and LUMO levels, respectively.

Within this model three generic scenarios are considered for the electron-vibration coupling $\tilde{M}$ in the energy basis, namely

$$\tilde{M}_1 = U^\dagger M_1 U = \begin{pmatrix} m & 0 \\ 0 & m \end{pmatrix} \quad \text{(S4)}$$

$$\tilde{M}_2 = U^\dagger M_2 U = \begin{pmatrix} 0 & m \\ m & 0 \end{pmatrix} \quad \text{(S5)}$$

$$\tilde{M}_3 = U^\dagger M_3 U = \begin{pmatrix} m & 0 \\ 0 & -m \end{pmatrix} \quad \text{(S6)}$$

corresponding to vibrational coupling to the charges (eq. S4), to interlevel scattering (eq. S5), and to HOMO-LUMO gap modulation (eq. S6). The coupling strength is characterized by m.

In the spirit of the lowest-order expansion approach to inelastic transport,[3–6] the elastic transmission $T(E)$ and inelastic conductance correction $\Delta(E)$, which reflects the height of the steplike variation in $\mathrm{d}I/\mathrm{d}V$ spectra, can be explored as a function of the Fermi energy (denoted E in this context) and independent model parameters (m/t, γ_L/t, and γ_R/t) by evaluating the following matrix expressions in the site basis:

$$T(E) = \mathrm{Tr}\{\Gamma_L A_R\} \quad \text{(S7)}$$

$$\Delta(E) = \mathrm{Tr}\{G^\dagger \Gamma_L G[M A_R M + \frac{\mathrm{i}}{2}(\Gamma_R G^\dagger M(A_L + A_R)M - h.c)]\} \quad \text{(S8)}$$

(with Tr the trace). Here $G(E)$ is the retarded Green's function and $A_{L/R}(E) = G\Gamma_{L/R}G^\dagger$ the lead-resolved spectral function.

A numerical analysis of $T(E)$ and $\Delta(E)$ is presented in Figure S10 for the three different types of electron-vibration couplings. With M_1-type charge coupling (left column) and M_3-type gap coupling (right column), the sign change in $\Delta(E)$ follows the "1/2-rule"

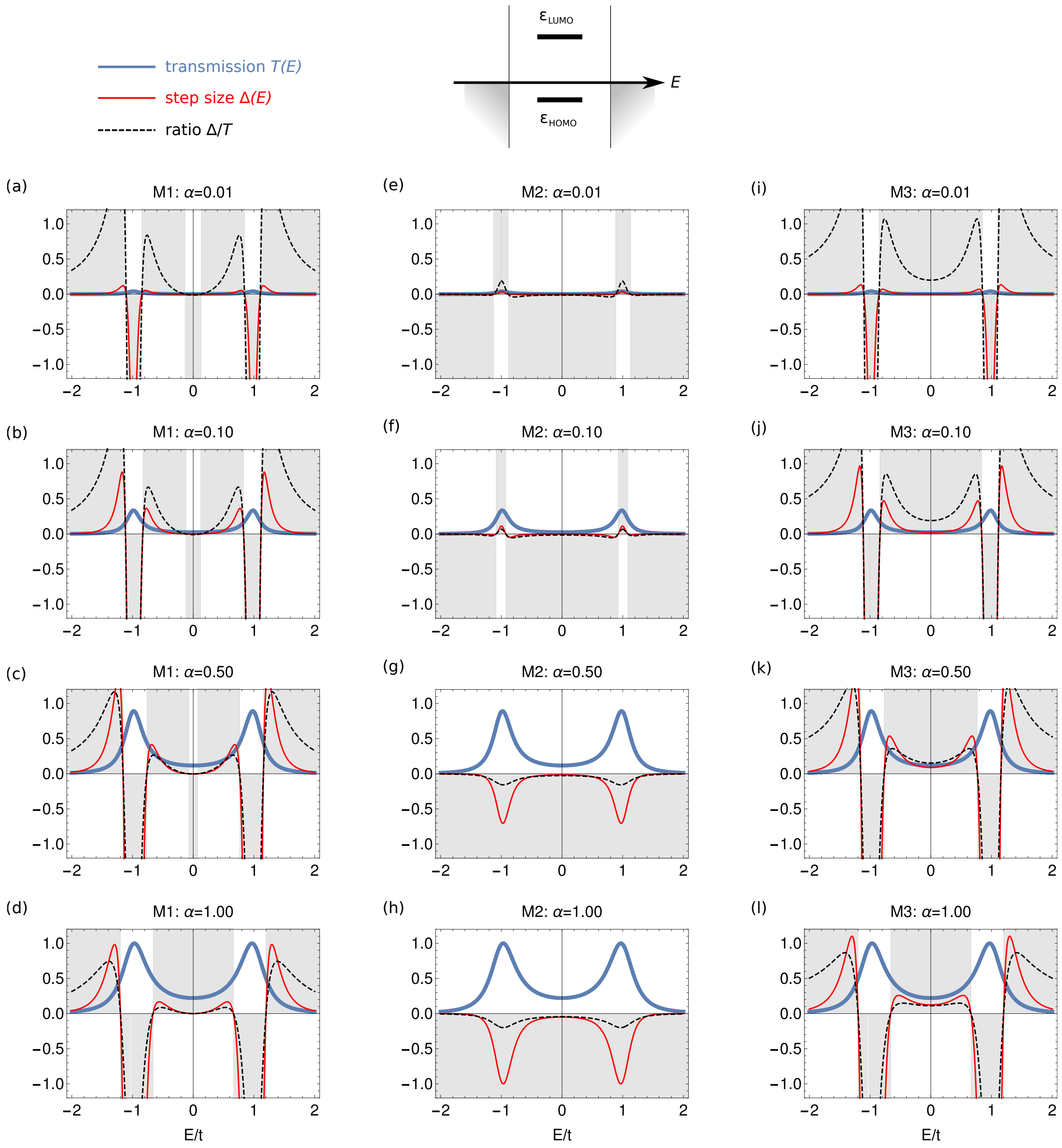

Figure S11: Evolution of the inelastic scattering signal from tunneling to contact for a simple two-level model (illustrated schematically at the top of the middle column). Each column corresponds to a fixed electron–vibration coupling of the form (a)–(d) M_1, (e)–(h) M_2 and (i)–(l) M_3. The asymmetry factor $\alpha = \gamma_L/\gamma_R$ is varied from top (tunneling) to bottom (contact) while maintaining $\gamma_L = t/2$ fixed. Blue curves show the elastic transmission $T(E)$; red and dashed black curves represent (in arbitrary units) the signal $\Delta(E)$ and ratio $(\Delta/T)(E)$, respectively; gray shaded areas highlight the sign of $\Delta(E)$.

reported previously for a single-level model,[3,4] namely conductance increases (decreases) for off-resonance (on-resonance) transport conditions at the onset of vibrational emission. Although a sign change also occurs mid-gap for M_1 near $E = 0$, the magnitude of this correction is minimal.

The situation is qualitatively different if one considers the M_2-type interlevel coupling as shown in the middle column of Figure S11. Under tunneling conditions ($\alpha \equiv \gamma_L/\gamma_R \ll 1$), the inelastic correction Δ shows the opposite behavior: conductance decreases (increases) for off-resonance (on-resonance) transport conditions. Further, with more symmetric coupling to the electrodes ($\alpha \approx 1$), the conductance correction is *always* negative.

These trends can be related with the findings reported in the article for the melamine junctions, namely a reduction of the IETS signal σ as the STM tip approaches contact distances to the molecule. Since σ measures the inelastic cross section per tunneling electron, a direct correlation of this signal with the ratio $(\Delta/T)(E)$ (dashed lines in Figure S11) displaying a similar behavior in the case of off-resonance transport together with M_1 or M_3 is suggested. An additional mechanism, that is the downshift of the LUMO resonance as the STM tip approaches the molecule (as observed in Figure S8), further tends to reduce the IETS signal. Finally, in the atomistic simulations reported here the electron-vibration coupling is not strictly constant as the tip approaches, potentially also affecting the signal strength.

References

(1) Ohto, T.; Rungger, I.; Yamashita, K.; Nakamura, H.; Sanvito, S. Ab Initio Theory for Current-Induced Molecular Switching: Melamine on Cu(001). *Phys. Rev. B* **2013**, *87*, 205439.

(2) Frederiksen, T. Inelastica (https://github.com/tfrederiksen/inelastica). **2020**.

(3) Paulsson, M.; Frederiksen, T.; Brandbyge, M. Modeling Inelastic Phonon Scattering in Atomic- and Molecular-Wire Junctions. *Phys. Rev. B* **2005**, *72*, 201101.

(4) Frederiksen, T.; Paulsson, M.; Brandbyge, M.; Jauho, A.-P. Inelastic Transport Theory from First Principles: Methodology and Application to Nanoscale Devices. *Phys. Rev. B* **2007**, *75*, 205413.

(5) Lü, J.-T.; Christensen, R. B.; Foti, G.; Frederiksen, T.; Gunst, T.; Brandbyge, M. Efficient Calculation of Inelastic Vibration Signals in Electron Transport: Beyond the Wide-Band Approximation. *Phys. Rev. B* **2014**, *89*, 081405.

(6) Paulsson, M.; Frederiksen, T.; Ueba, H.; Lorente, N.; Brandbyge, M. Unified Description of Inelastic Propensity Rules for Electron Transport through Nanoscale Junctions. *Phys. Rev. Lett.* **2008**, *100*, 226604.
